# Case Report: Membranous/cytoplasmic Ki-67 staining and PAX8-GLIS3 fusion: defining the clinicopathological spectrum of hyalinizing trabecular tumor to optimize patient management

**DOI:** 10.3389/fmed.2026.1764079

**Published:** 2026-02-16

**Authors:** Haining Huang, Ran Zhao, Liman Zhang, Shujun Ren, Jianli Liu, Dandan Yang, Junjun Zhang, Renya Zhang, Shuai Chen, Lei Li

**Affiliations:** 1 Department of Clinical Pharmacy, Affiliated Hospital of Jining Medical University, Jining, China; 2 Department of Pathology, Affiliated Hospital of Jining Medical University, Jining, China

**Keywords:** *BRAF*, clinicopathological feature, hyalinizing trabecular tumor, Ki-67, papillary thyroid carcinoma, *PAX8-GLIS3* fusion

## Abstract

Hyalinizing trabecular tumor (HTT) is a rare thyroid neoplasm with an excellent prognosis, yet its morphological resemblance to malignancies such as papillary thyroid carcinoma (PTC) often complicates its diagnosis. To characterize the clinicopathological, immunohistochemical, and molecular profiles of HTT, this study analyzed 21 HTT cases and 10 PTC cases, and the findings were supplemented by a literature review. The HTT cohort included 18 female and 3 male patients, with a mean age of 51 years. Cytologically, HTT can be distinguished from PTC by its distinctive membranous/cytoplasmic Ki-67 staining pattern and the presence of the *PAX8-GLIS3* gene fusion and occasional polyploid cells. Histologically, the tumors were well-demarcated and exhibited trabecular or organoid growth, eosinophilic to granular cytoplasm, paranuclear yellow inclusions, and hyalinized stroma. Immunohistochemically, HTT consistently expressed TG, TTF1, and CD56, while Ki-67 showed a unique membranous/cytoplasmic distribution. Molecular profiling identified no *KRAS*, *NRAS*, *BRAF*, or *PIK3CA* mutations. However, *PAX8-GLIS3* fusion was detected in all HTT cases, a finding absent from PTC. The use of Ki-67 immunohistochemistry or *PAX8-GLIS3* testing on cytological specimens can aid in definitive diagnosis and prevent unnecessary surgery. Thus, an integrated approach combining cytological, histological, immunohistochemical, and molecular data is essential for the accurate diagnosis and optimal clinical management of HTT.

## 1 Introduction

Hyalinizing trabecular tumor (HTT) is a rare neoplasm originating from the thyroid follicular epithelium. In 1987, Carney et al. initially provided a detailed description of HTT (1) HTT is characterized primarily by a trabecular growth pattern and prominent intratrabecular hyalinization. In this study, we conducted an in-depth analysis of HTT’s clinicopathological characteristics, immunophenotype, diagnosis, differential diagnosis, and molecular genetics by retrospectively reviewing 21 HTT cases and integrating relevant literature. The ultimate aim of the study is to improve the accuracy of HTT pathological diagnosis through a multifaceted analysis and to help prevent overtreatment.

## 2 Materials and methods

### 2.1 Materials

This study included 20 cases diagnosed as HTT in the Department of Pathology of the Affiliated Hospital of Jining Medical University from 2012 to 2025 and one case from the People’s Hospital of Jiaxiang County. Among them, there were 18 female patients and 3 male patients, with an age range of 24–72 years and an average age of 51 years. Twenty patients sought medical attention for imaging findings or neck masses, while one patient presented with hoarseness. Ten additional patients were pathologically confirmed to have papillary thyroid carcinoma (PTC) at our institution, despite initial cytological features partially mimicking HTT. This was a retrospective study and was approved by the Ethics Committee of the Affiliated Hospital of Jining Medical University.

### 2.2 Methods

All specimens were fixed in 10% neutral formalin, followed by routine dehydration, paraffin embedding, and sectioning. HE staining, immunohistochemistry, and gene detection were then performed sequentially. All sections were reviewed and validated by at least two attending pathologists.

For immunohistochemical staining, the EnVision method was adopted, with diaminobenzidine (DAB) as the chromogenic reagent and hematoxylin for counterstaining. All selected antibodies and kits were ready-to-use products. Except for Ki-67, which was purchased from Gene Tech (Shanghai, China), the remaining antibodies were purchased from Maixin Biotechnology (Fuzhou, China). Specifically, the positive expression of thyroglobulin (TG) (Clone No. MX131), cytokeratin 19 (CK19) (Clone No. MX054), galectin-3 (Clone No. MX060), and calcitonin (CT) (Clone No. MXR059) was localized in the cytoplasm; thyroid transcription factor-1 (TTF-1) (Clone No. MX011) was positively expressed in the nucleus; CD56 (Clone No. MX039) showed positive localization on the cell membrane; and Ki-67 (Clone No. MIB-1) exhibited positive staining on the cell membrane/cytoplasm (nuclear positivity was defined as negative).

The Sanger sequencing method was used to detect gene mutations in *KRAS* (exons 2 and 3), *NRAS* (exons 2, 3, and 4), *BRAF V600E*, and *PIK3CA* (exons 9 and 20). DNA was extracted from paraffin-embedded tissues using a kit from Tiangen Biotech (Beijing, China), and mutations were detected with the KNBP kit from Xiamen AmoyDx (Xiamen, China). Fluorescence *in situ* hybridization (FISH) was performed to detect *PAX8-GLIS3* gene fusion using a FISH kit supplied by Kanglu Biotechnology (Wuhan, China).

## 3 Results

### 3.1 Clinical data

**Figure 1:**
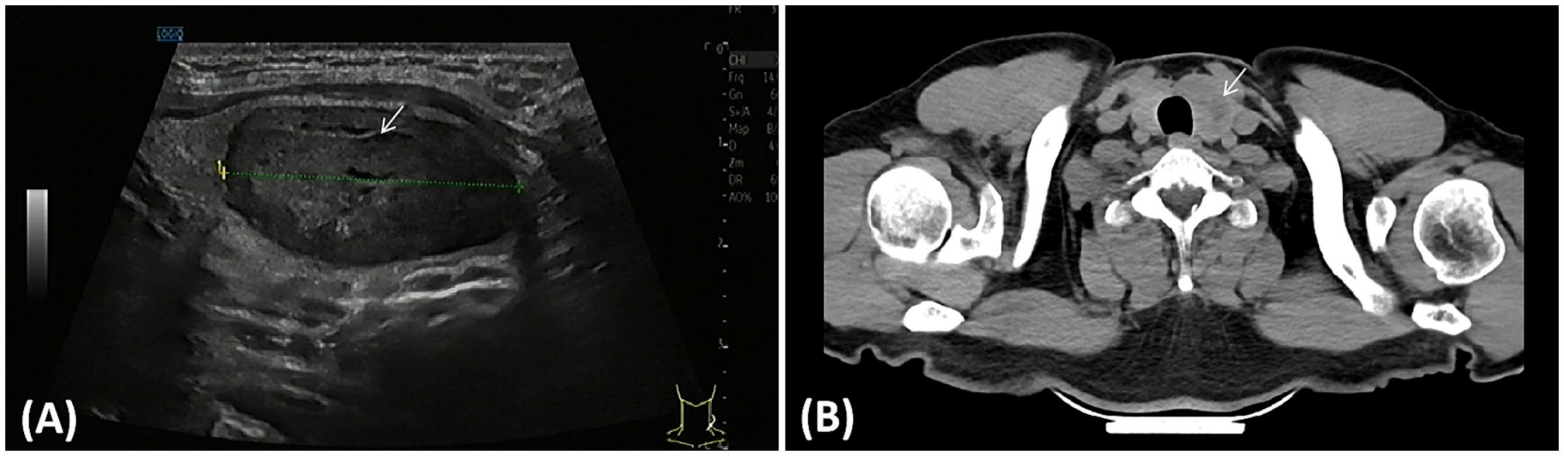
Imaging findings of the hyalinizing trabecular tumor. **(A)** Ultrasound demonstrated a solitary cystic-solid nodule in the middle and lower part of the left lobe, mainly solid and hypoechoic (arrow). **(B)** CT reveals an enlarged left lobe with a low-density nodule. The nodule is irregular in shape, has an ill-defined boundary, and compresses the trachea, causing its deviation to the right (arrow).

Of the 21 HTT cases, 14 were located in the left thyroid lobe and 7 in the right thyroid lobe. Ultrasound examination showed 12 hypoechoic solid nodules and 9 cystic-solid mixed nodules, with the solid components of these mixed nodules predominantly hypoechoic (Figure 1A). Computed tomography (CT) scans primarily demonstrated low-density nodular shadows (CT values 18–43 HU), local punctate calcifications, and relatively indistinct boundaries (Figure 1B). Laboratory examinations showed that, in 16 cases, thyroid function parameters—including thyroxine, triiodothyronine, thyroid-stimulating hormone, free triiodothyronine, and free thyroxine—anti-thyroglobulin antibody (TGAb), and anti-thyroid peroxidase antibody (TPOAb) were within the normal range. In five cases, TPOAb levels were elevated (203,000 IU/mL and 142,000 IU/mL, respectively). In one case, free thyroxine levels were decreased (9.89 pmol/L), and in another case, thyroid-stimulating hormone levels were elevated (4.83 mIU/L). Eight patients underwent subtotal thyroidectomy, seven patients received unilateral thyroidectomy accompanied by isthmectomy, and four patients had hemithyroidectomy. In the remaining two cases, thyroid fine-needle aspiration (FNA) showed atypical cells suggestive of HTT; therefore, surgical resection was not performed, and close follow-up was advised. All 21 HTT cases were followed for 5–162 months, with no recurrence or metastasis observed (Table 1).

**Table 1:** Clinical information and pathological features of 21 patients with HTT.

Case	Sex	Age	Presentation	Size(cm)	US	Surgery	Follow-up (m)
1	F	50	Lt neck mass	2	Multiple cystic, solid and mixed nodules: TI-RADS 3	Subtotal thyroidectomy	Well at 162 m
2	F	69	Rt neck mass	3.5	Multiple cystic, solid and mixed nodules: TI-RADS 3	Subtotal thyroidectomy	Well at 125 m
3	F	41	Lt thyroid lobe nodule in PE	0.6	Hypoechoic solid nodule with calci-fication: TI-RADS 4	Subtotal thyroidectomy	Well at 124 m
4	F	52	Lt neck mass	1.8	Multiple cystic, solid nodule: TI-RADS 4	Subtotal thyroidectomy	Well at 80 m
5	M	33	Rt neck mass	1.8	Hypoechoic solid mass: TI-RADS 4	Follow-up with-out surgery	Well at 80 m
6	F	62	Rt neck mass	2.0	Multiple cystic, solid nodule: TI-RADS 4	Subtotal thyroidectomy	Well at 72 m
7	F	34	Lt thyroid lobe nodule in PE	2.0	Hypoechoic solid mass: TIRADS 4	Lt Hemithyroidec tomy	Well at 71 m
8	F	51	Rt neck mass	2.5	Hypoechoic solid mass: TI-RADS 4	Follow-up with-out surgery	Well at 66 m
9	F	38	Lt thyroid lobe nodule	5.0	Mixed echogenic mass: TI-RADS 3	Lt Hemithyroidec tomy	Well at 61 m
10	F	58	Lt neck mass	0.3	Multiple solid nodule: TI-RADS 3	Subtotal thyroidectomy	Well at 47 m
11	M	55	Lt lobe and isthmus nodule, hoarse	1.5	Cystic solid nodules: TI-RADS 3	Lt lobe & isthmus resection	Well at 34 m
12	F	64	Lt thyroid lobe nodule	6.0	Hypoechoic solid nodule: TI-RADS 3	Subtotal thyroidectomy	Well at 32 m
13	F	61	Lt neck mass	4.0	Isohypoechoic solid nodule: TI-RADS 4a	Subtotal thyroidectomy	Well at 30 m
14	F	52	Lt thyroid lobe nodule in PE	0.7	Hypoechoic solid nodule: TI-RADS 4	Lt lobe & isthmus resection	Well at 30 m
15	M	53	Lt thyroid lobe nodule in PE	3.5	Cystic solid Nodules: TIRADS 3	Lt lobe & isthmus resection	Well at 19 m
16	F	61	Rt thyroid lobe nodule in PE	1.0	Hypoechoic solid nodule: TI-RADS 3	Rt Hemithyroidectomy	Well at 18 m
17	F	51	Lt thyroid lobe nodule in PE	3.5	Cystic solid nodules: TI-RADS 3	Lt Hemithyroidectomy	Well at 17 m
18	F	45	Rt neck mass	4.5	Isohypoechoic solid nodule: TI-RADS 4a	Rt lobe & isthmus resection	Well at 16 m
19	F	61	Lt neck mass	5.0	Isohypoechoic solid nodule: TI-RADS 4a	Lt lobe & isthmus resection	Well at 13 m
20	F	24	Rt neck mass	1.1	Isohypoechoic solid nodule: TI-RADS 4a	Rt lobe & isthmus resection	Well at 7 m
21	F	72	Rt neck mass	2.7	Isohypoechoic solid nodule: TI-RADS 3	Rt lobe & isthmus resection	Well at 5 m

F, female; M, male; cm, centimeters; US, ultrasonography; m, months; Rt, right; Lt, left; NA, unavailable.

### 3.2 Macroscopic appearance

The majority of the tumors presented as solid nodules or lobulated masses. Among them, 18 cases had a complete capsule, 2 cases had an incomplete capsule, and, in 1 case, the boundary of the capsule was unclear. The smallest tumor had a diameter of 0.3 cm, and the largest one measured 6.0×4.5×4.0 cm. The cut surface of all tumors was grayish-red or grayish-yellow, with a small part of the surface being grayish-white. The texture was soft or moderate.

### 3.3 Microscopic examination

**Figure 2:**
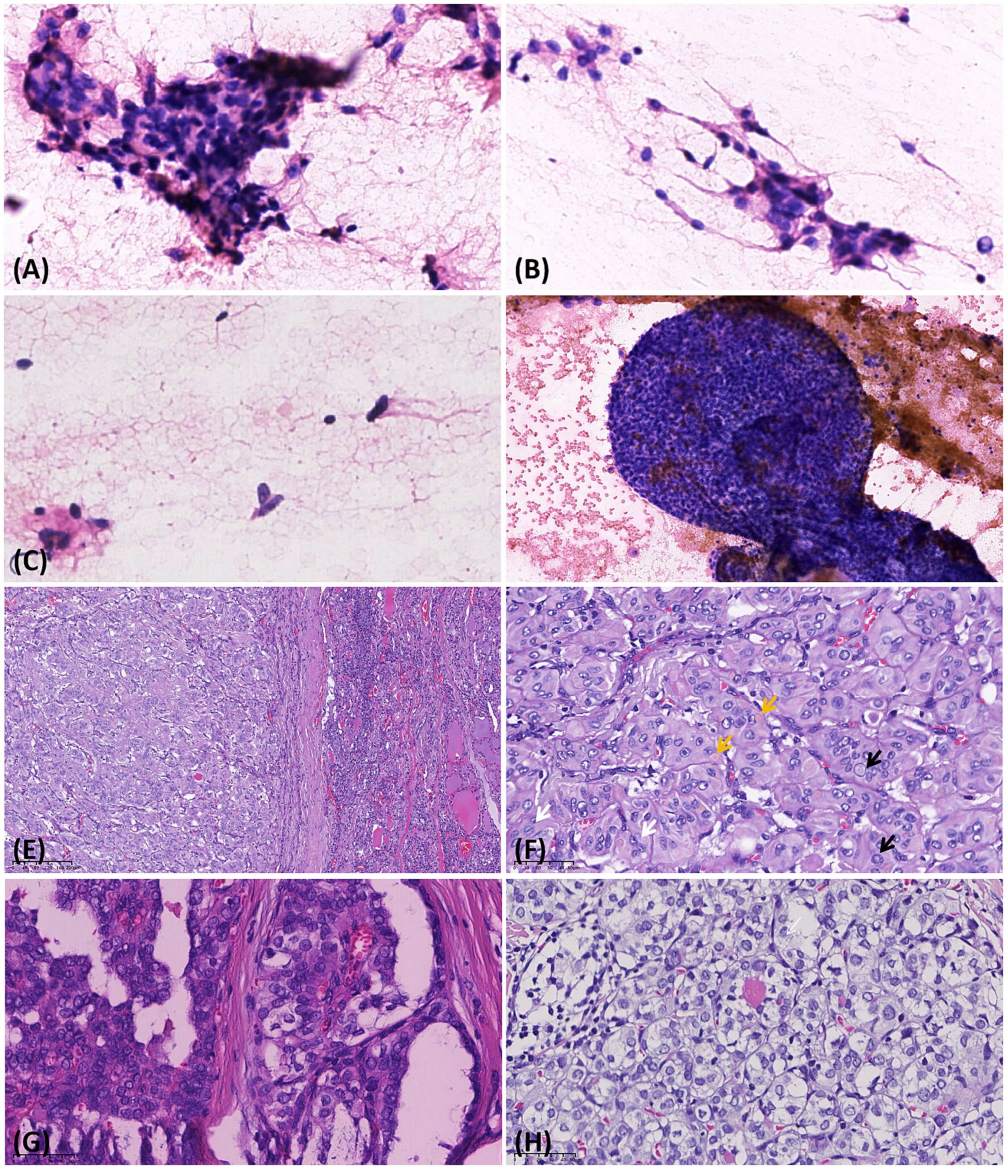
Cytological and histological findings of the hyalinizing trabecular tumor and papillary thyroid carcinoma. **(A,B)** Fine-needle aspiration cytology shows follicular cell clusters with enlarged, polygonal nuclei, fine granular chromatin, intranuclear pseudoinclusions, and mild nuclear atypia (HE 400×). **(C)** Focal presence of atypical megakaryocytes is identified in the HTT (fine-needle aspiration) cytology. **(D)** PTC exhibited more prominent nuclear atypia and was frequently accompanied by distinct papillary structures (HE 200×). **(E)** Under low-magnification microscopy, HTT typically manifests as a solitary neoplasm with distinct boundaries, featuring a trabecular architectural pattern (HE 100×). **(F)** The tumor cells exhibit a band-like morphology, featuring elongated nuclei oriented perpendicular to the long axis of the trabeculae. Occasional nuclear pseudoinclusions (black arrow) and nuclear grooves (white arrow) are observed. In contrast to PTC, HTT frequently displayed cytoplasmic yellow bodies (yellow arrow; HE 400×). **(G)** The classic subtype, the most prevalent variant of PTC, is characterized by papillary architectures and also exhibits intranuclear pseudoinclusions and nuclear grooves (HE 400×). **(H)** The second most common variant is the follicular variant of PTC, which is occasionally morphologically indistinguishable from HTT on routine HE staining (HE 400×).

Eight cases underwent fine-needle aspiration cytology (FNAC) before surgery. FNAC showed various clusters of follicular cells with enlarged polygonal or fusiform nuclei and fine granular chromatin (Figure 2A). Some cells exhibited nuclear grooves and intranuclear pseudoinclusions (Figure 2B), and focal atypia in megakaryocytes was noted on HTT aspiration cytology (Figure 2C). The overall nuclear atypia of HTT was mild, and the tumors in six cases were diagnosed as atypia of undetermined significance (AUS). Meanwhile, one case (case 15) was misdiagnosed as PTC. Although PTC exhibited similar cytological features, it demonstrated more obvious atypia and was accompanied by distinct papillary structures (Figure 2D). Following comprehensive evaluation, 19 cases were surgically resected, while 2 cases were managed with close follow-up. The microscopic morphology of the 19 cases of HTT in this group was largely uniform. The tumor had a clear boundary, and a thin fibrous capsule was visible. Under low-magnification microscopy, HTT typically presents as a solitary neoplasm with well-defined borders and a trabecular architectural pattern, with interstitial hyalinization identified (Figure 2E). The tumor cells were oval and moderately large, with abundant cytoplasm that was eosinophilic or transparent, finely granular, and partially amphophilic. The tumor nuclei were elongated, with obvious membrane irregularities, forming nuclear grooves and intranuclear pseudoinclusions, and were arranged perpendicular to the long axis of the trabeculae (Figure 2F). Scattered, small, pale-yellow, round bodies were observed around the nuclei (Figure 2F), along with perivascular hyalinization, fibrosis, and cytoplasmic hyalinization. Notably, PTC encompasses multiple subtypes, with the classic subtype (Figure 2G) and follicular subtype (Figure 2H) being the most prevalent. These subtypes are occasionally morphologically indistinguishable from HTT, highlighting the need for immunohistochemical and molecular detection assays for aiding differential diagnosis. In HTT specimens, nuclear grooves were observed in 12 cases, intranuclear pseudoinclusions in 13 cases, and small nucleoli were present. Mitotic figures were observed in one case, and psammoma bodies were noted in two cases, exhibiting nuclear features similar to those of PTC.

### 3.4 Immunohistochemical staining

**Figure 3:**
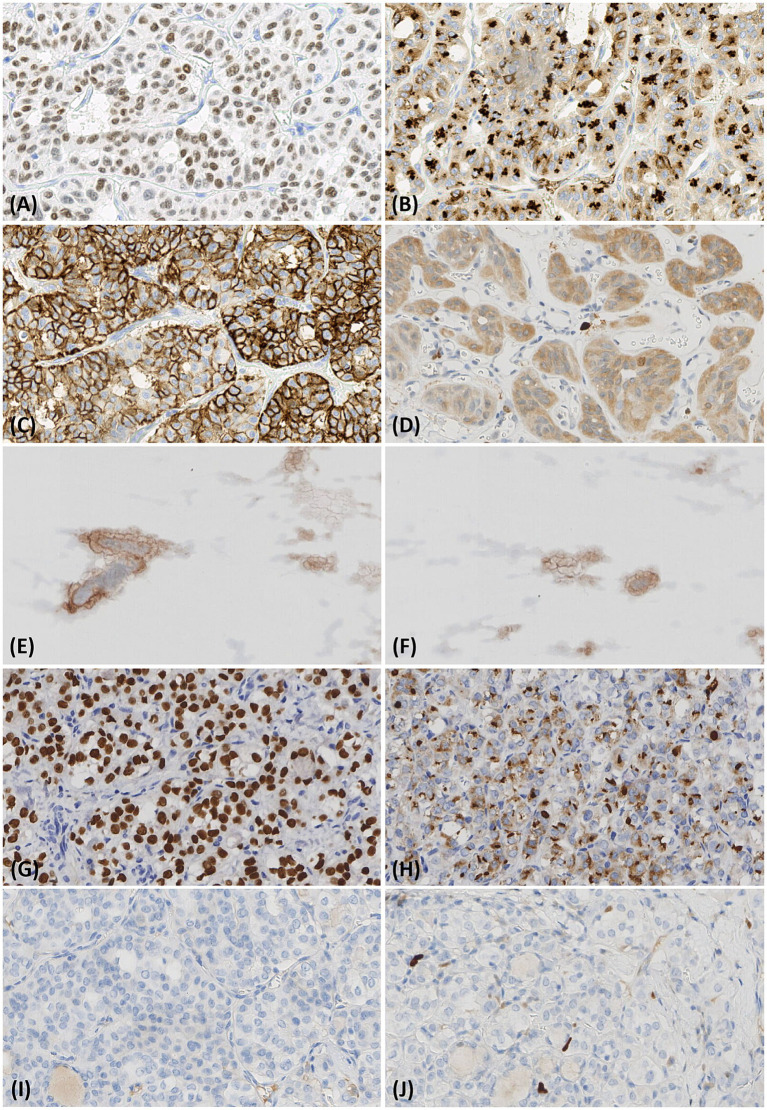
Immunohistochemical findings of the hyalinizing trabecular tumor and papillary thyroid carcinoma. **(A–C)** Immunohistochemical staining revealed that TTF1, TG, and CD56 were strongly positive in the HTT (IHC 400×). **(D)** The tumor cells show membranous/cytoplasmic immunoreactivity of Ki-67 (MIB-1; IHC 400×). **(E, F)** FNA cells also show membranous/cytoplasmic immunoreactivity of Ki-67 (MIB-1; IHC 400×). **(G–I)** In PTC, immunohistochemical staining demonstrated positive expression of TTF1 and TG, whereas CD56 expression was negative (IHC 400×). **(J)** PTC tumor cells show nuclear positivity for Ki-67 (MIB-1; IHC 400×).

In HTT cases, immunohistochemical staining revealed high positivity for TTF1 (18/19, 95%, Figure 3A), TG (17/19, 89%, Figure 3B), and CD56 (18/19, 95%, Figure 3C). By contrast, focal positivity was observed for galectin-3 (11/19, 58%) and cytokeratin 19 (CK19; 4/19, 21%) in a minority of cases. However, Ki-67 showed characteristic positive staining in the cell membrane/cytoplasm (Figure 3D). Ki-67 analysis of FNA cells was also positive, consistent with the histological findings (Figures 3E,F). The specific results are shown in Table 2. The typical immunophenotype of PTC was as follows: positive for TTF1 (Figure 3G) and TG (Figure 3H), negative for CD56 (Figure 3I), and nuclear positivity for Ki-67 (Figure 3J) with a labeling index of 2–5%.

**Table 2:** Immunohistochemical and molecular genetic findings in 21 HTT cases and 10 PTC cases.

Case	TTF1	CD56	TG	CK19	Galactin3	Ki-67	PAX8-GLIS3 fusion
1	+	+	+	−	−	Cyt/Mem +	UA
2	+	+	+	−	Focal +	Cyt/Mem +	−
3	+	+	+	−	−	Cyt/Mem +	+
4	+	+	+	−	Partial +	Cyt/Mem +	+
5	UA	UA	UA	UA	UA	Cyt/Mem +	+
6	+	+	+	−	−	Cyt/Mem +	+
7	−	+	+	+	Partial +	Cyt/Mem +	+
8	UA	UA	UA	UA	UA	Cyt/Mem +	+
9	+	+	+	Focal +	−	Cyt/Mem +	+
10	+	−	−	−	−	−	+
11	+	Partial +	+	+	+	Cyt/Mem +	−
12	+	+	+	−	+	Cyt/Mem +	−
13	+	+	+	−	Partial +	Cyt/Mem +	+
14	+	+	+	Focal +	+	Cyt/Mem +	UA
15	+	+	+	−	−	Cyt/Mem +	+
16	+	+	−	−	Partial +	Cyt/Mem +	−
17	+	+	+	−	Focal +	Cyt/Mem +	+
18	+	+	+	−	Partial +	Cyt/Mem +	+
19	+	+	+	−	−	Cyt/Mem +	+
20	+	+	+	−	−	Cyt/Mem +	+
21	+	+	+	−	+	Cyt/Mem +	+
22	+	−	+	+	+	Nuc +	−
23	+	−	+	+	+	Nuc +	−
24	+	−	+	+	+	Nuc +	−
25	+	−	+	+	+	Nuc +	−
26	+	−	+	+	+	Nuc +	−
27	+	−	+	+	Partial +	Nuc +	−
28	+	−	+	Partial +	+	Nuc +	−
29	+	−	+	+	+	Nuc +	−
30	+	−	+	+	+	Nuc +	−
31	+	−	+	+	+	Nuc +	−

Cyt, cytoplasm; Mem, membrane; Nuc, nuclear; UA, unavailable.

### 3.5 Molecular analysis

**Figure 4:**
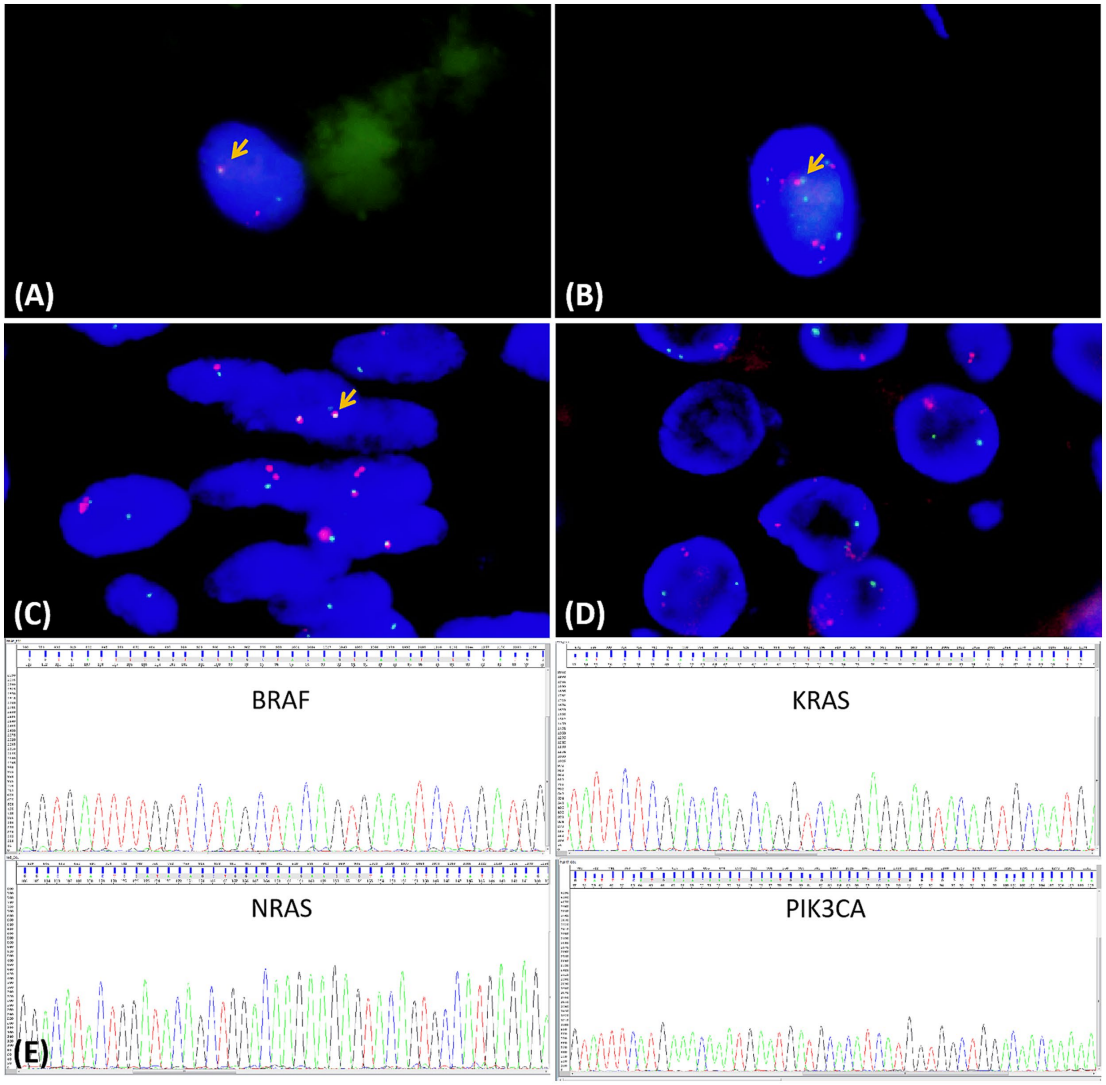
Molecular findings of the hyalinizing trabecular tumor and papillary thyroid carcinoma. **(A)** FISH staining of fine-needle aspiration cells at HTT showed *PAX8-GLIS3* fusion (yellow arrow). **(B)** Atypical megakaryocytes in FNA cytology harbored *PAX8-GLIS3* fusion with concurrent *PAX8/GLIS3* copy number gains. **(C,D)** By FISH for the *PAX8-GLIS3* gene fusion, red-green signal fusion was seen in HTT (yellow arrow), but not in PTC. **(E)** No mutations in KRAS, NRAS, BRAF, or PIK3CA were detected in HTT specimens via Sanger sequencing.

FNA cells from HTT demonstrated *PAX8-GLIS3* gene fusion (Figure 4A), whereas no such fusion was detected in PTC. The atypical megakaryocytes observed in FNAC also demonstrated *PAX8-GLIS3* fusion, along with an intriguing copy number gain of both *PAX8* and *GLIS3* (Figure 4B). In HTT, 15 cases (78.9%) were positive for the *PAX8-GLIS3* gene fusion detected by FISH, whereas all PTC cases were negative (Figures 4C,D), and the specific results are shown in Table 2. No mutations in the *KRAS*, *NRAS*, *BRAF*, and *PIK3CA* genes were detected in 19 HTT cases using the Sanger sequencing method (Figure 4E). Gene detection was not performed in cases 1 and 14. In contrast, *BRAF* gene mutations were detected in eight PTC cases, and *NRAS* mutations were detected in two PTC cases.

## 4 Discussion

Carney et al. first reported a relatively infrequent thyroid neoplasm, naming it HTT due to its characteristic trabecular architecture and interstitial hyalinization. Notably, among 13 reported HTT cases, no recurrence or metastasis occurred over a 10-year average follow-up period (1). In 2008, Carney et al. conducted a follow-up study on 119 HTT patients, and only a single patient presented with vascular and capsular invasion, as well as pulmonary metastasis (2). Consequently, it was postulated that the majority of HTT cases are benign. However, malignant biological behaviors can manifest in a minority of cases (3, 4). Therefore, HTT is currently regarded as a tumor type with low malignant potential. All 21 cases of HTT in this cohort exhibited features of benign neoplasms. They showed no evidence of vascular structure or capsule invasion, and no recurrence or metastasis occurred during the follow-up period. Admittedly, the follow-up duration for some of our cases was relatively short, and we will continue to track these cases to further improve the prognostic data. Clinically, HTT typically presents as an asymptomatic neck mass—frequently detected during routine physical examinations—and is more prevalent in adult female patients (5, 6). On ultrasonography, it usually appears as an oval or round hypoechoic solid nodule with smooth margins (7). Perinodular or intranodular vascularity may also be present, and cystic alterations may occur (5, 8). The etiopathogenesis of HTT remains elusive, but it has been reported to have a certain correlation with chronic lymphocytic thyroiditis (9). Among the 21 HTT cases, ultrasound predominantly revealed solid hypoechoic nodules, with a few cases presenting heterogeneous echoes. In five cases, HTT co-existed with chronic lymphocytic thyroiditis, which also confirms the findings reported in the literature.

In FNAC, the majority of HTT cases are classified into the indeterminate Bethesda category (10). Due to the presence of nuclear features analogous to those of PTC, such as intranuclear pseudoinclusions and nuclear grooves, HTT is readily confounded with PTC (7, 11–13). Additionally, PTC can also manifest with focal hyalinization or trabecular growth patterns. In the majority of our cases, cytology revealed that the proliferative follicular epithelial cells had eosinophilic alterations, the nuclei were slightly eccentric, some were conspicuously enlarged, pseudoinclusions were discernible, and nuclear grooves and nucleoli were not readily observable. Five of eight cases were considered atypical cellular lesions of undetermined significance, and a right hemithyroidectomy was performed. However, two of the eight cases avoided surgery after comprehensive evaluation and were managed with close follow-up. Case 15 was reported as PTC based on cytological features similar to those of PTC, and a left lobectomy with the isthmus was performed. Additionally, mild atypia in megakaryocytes was observed on HTT aspiration cytology. Therefore, achieving an accurate and effective cytological diagnosis of HTT is of paramount importance for guiding appropriate surgical management and avoiding unnecessary total thyroidectomy. Choi et al. proposed that when FNA suggests PTC but ultrasonography does not display malignant features, HTT should be considered (7). Currently, the key cytological features for the diagnosis of HTT primarily encompass a hemorrhagic background; cells arranged individually or in clusters radiating around hyalinized substances; and cells that are round, polygonal, or elongated, with eosinophilic cytoplasm, a low nucleus-to-cytoplasm ratio, and indistinct cell boundaries. The nuclei are enlarged and eccentric and are oval or spindle-shaped, with intranuclear pseudoinclusions and nuclear grooves, as observed in our cases. In addition, fibrosis/hyaline matrices and dense mucinous masses similar to colloid may be present, and yellow bodies can also emerge in the cytoplasm (6, 11, 14). Furthermore, the hyalinized substances within the trabeculae of HTT, which are similar to amyloid staining, may also be misdiagnosed as medullary thyroid carcinoma (MTC) (15). However, the chromatin of MTC nuclei is mostly “salt-and-pepper” like, with a thin nuclear membrane, loosely arranged, and a clean background.

Grossly, HTT preponderantly presents as a single solid nodule. The cut surface is grayish-red or grayish-yellow, medium-textured, and encapsulated, with clear separation from normal tissues. In this study’s 21 HTT cases, 10 cases presented as single solid nodules, some with calcifications. One case was adjacent to the capsule with an indistinct boundary. The remaining 10 cases mostly occurred concomitantly with PTC or nodular goiter. Five cases were complicated with Hashimoto’s thyroiditis and presented as multinodular masses grossly, with a grayish-white, tough or hard texture, and some had incomplete capsules.

Microscopically, HTT has a clear demarcation and may possess a fibrous capsule arranged in a trabecular or fascicular pattern. The tumor cells are polygonal or spindle-shaped, with abundant cytoplasm that is eosinophilic or hyaline. The nuclei display varying degrees of pleomorphism, arranged perpendicular to the long axis of the trabeculae. The chromatin is in fine granules, and intranuclear pseudoinclusions and nuclear grooves can be observed. Unique yellow bodies are commonly found around the nuclei, while psammoma bodies are rare, and mitotic figures are sporadically observed. Trabeculae contain abundant eosinophilic hyalinized substances. These substances tested positive for periodic acid-Schiff and type IV collagen staining but negative for Congo red staining, helping to distinguish HTT from MTC. All cases in this study showed typical HTT features: trabecular structure and interstitial hyalinization. Abundant eosinophilic hyalinized substances in trabeculae are a histological hallmark of HTT. Hino et al. posited that this hyaline matrix is related to calcium deposition (16). Special von Kossa staining revealed that, in all HTT cases, a large number of minute black dots were shown around the blood vessels in the hyaline matrix, while in most PTC cases, it was negative, and only a few showed linear or circular black lines in the fibrous capsule or fibrous lesions. This might be a valuable diagnostic clue for HTT. Furthermore, an immunohistochemical analysis reveals distinct features of HTT, including characteristic cell membrane and cytoplasmic staining of Ki-67 (MIB-1), which aids in its differentiation from other thyroid neoplasms (17). Additionally, HTT tumor cells exhibit consistent immunopositivity for TG and TTF1, focal expression of CK19 and galectin-3, and negativity for calcitonin and chromogranin. This immunoprofile effectively distinguishes HTT from PTC and MTC. In the HTT cases in this study, the immunohistochemical results demonstrated positive expressions of TG, TTF1, CD56, etc. Among these cases, 20 exhibited positive membranous/cytoplasmic expression of Ki-67 (MIB-1), whereas all PTC cases displayed nuclear expression, with the labeling index ranging from 2 to 5%. However, one Ki-67-negative case exhibited typical HTT histological features and showed positive *PAX8-GLIS3* gene fusion by FISH. This discrepancy may result from technical variables such as dewaxing efficiency, antigen retrieval temperature, or protein blocking during immunohistochemistry. Ki-67 staining outcomes appear particularly dependent on the staining platform, as exemplified by the Dako Autostainer Link 48, which will be used for the prospective validation in follow-up studies. Therefore, when cytology suggests HTT, a definitive diagnosis can be made by combining Ki-67 immunohistochemical staining or *PAX8-GLIS3* molecular detection to avoid surgical resection, as exemplified by our cases 5 and 8, who have remained healthy with over 5 years of follow-up.

Another similarity between HTT and PTC is that HTT was once reported to harbor RET-PTC gene mutations and was regarded as the “hyaline trabecular” variant of papillary carcinoma (18). However, in subsequent investigations, the characteristic *BRAF* and *RAS* gene mutations of PTC were not detected (10, 19). Sheu et al. detected the expression patterns of five miRNAs (146b, −181b, −21, −221, and −222) known to be upregulated in PTC by reverse transcription-polymerase chain reaction (RT-PCR) in HTT. The results indicated that, contrary to PTC, all miRNAs were downregulated in HTT tissues (20). Nikiforova et al. discovered that *GLIS* gene rearrangement, especially *PAX8-GLIS3* gene rearrangement, is one of the molecular biological markers of HTT (21). Moreover, in tumor tissues with PAX8-GLIS3 gene rearrangement, genes related to the extracellular matrix, including most collagen genes such as COL4A1, COL5A3, and COL5A2, were significantly augmented. This may explain the excessive production and deposition of type IV collagen and other collagens in HTT, resulting in extensive hyalinization, whereas *GLIS* gene rearrangement has not been detected in PTC (21–23). In this study, *PAX8-GLIS3* gene fusion was identified by FISH in 15 of 19 HTT cases (78.9%). The remaining four cases were hypothesized to harbor *PAX8-GLIS1* fusion, though further validation is required. Notably, our study reveals that atypical polyploid cells identified in HTT FNAC uniformly exhibited *PAX8-GLIS3* fusion, accompanied by concurrent *PAX8/GLIS3* copy number gains, a distinctive genetic signature not previously reported in other thyroid neoplasms. Meanwhile, these megakaryocytes also exhibit Ki-67 positivity in the cell membrane and cytoplasm, which further supports that they are tumor cells of HTT. This finding strongly supports a unique association between this molecular alteration and aberrant megakaryocytic differentiation in HTT. Mechanistically, the *PAX8-GLIS3* fusion places *GLIS3* exons 3–11 under the control of the thyroid-specific *PAX8* promoter, leading to increased *GLIS3* copy number and consequent protein overexpression (24, 25). The *PAX8-GLIS3* fusion protein may drive polyploidization through dysregulation of cell cycle checkpoints or transcriptional disruption, ultimately resulting in chromosomal segregation errors (26). This results in constitutive *GLIS3* activation, which confers cellular properties essential for tumorigenesis. Collectively, these data support a model wherein *PAX8-GLIS3* fusion promotes thyroid tumorigenesis by inducing tetraploidization and megakaryocyte-like transdifferentiation, unveiling a previously unrecognized oncogenic axis in HTT. In addition, no other genetic alterations, including *KRAS*, *NRAS*, *BRAF*, and *PIK3CA* mutations, were detected in the 19 cases of HTT, which was congruent with the above literature reports. Therefore, differences in molecular biological markers confirm that HTT is distinct from PTC. Preoperative genetic testing of thyroid nodules helps distinguish HTT from PTC, MTC, and other thyroid tumors.

## 5 Conclusion

In conclusion, the cytological diagnosis of HTT is susceptible to misinterpretation due to its relative rarity. In such scenarios, Ki-67 immunohistochemical staining or *PAX8-GLIS3* molecular testing can be applied to cytological specimens to confirm the diagnosis. Moreover, HTT generally demonstrates low malignant potential and a favorable prognosis, making close clinical follow-up a viable alternative to surgical resection. In this study, we conducted a comprehensive multifaceted analysis of a large cohort of HTT cases. Our results demonstrate that integrating cytopathological, histological, immunohistochemical, molecular genetic, and imaging characteristics enables clinicians to accurately diagnose HTT, thereby effectively avoiding overtreatment.

## Data Availability

The raw data supporting the conclusions of this article will be made available by the authors, without undue reservation.
